# Beyond Hamstring Shortness: Continuous Flexibility Is Associated With Balance and Stability but Not Sensory Measures—A Cross‐Sectional Study

**DOI:** 10.1155/tswj/5729491

**Published:** 2026-08-13

**Authors:** Alp Özel, Şeriban Şeşen, Senanur Akıncı

**Affiliations:** ^1^ Department of Physiotherapy and Rehabilitation, Faculty of Health Sciences, Bolu Abant İzzet Baysal University, Bolu, Turkey

**Keywords:** dynamic balance, hamstring flexibility, postural stability, two-point discrimination, Y Balance Test proprioception

## Abstract

**Background:**

Hamstring flexibility may influence postural control through its effects on movement mechanics and lower extremity function. However, whether flexibility is associated equally with motor and sensory components of postural control remains unclear. In addition, previous studies have predominantly relied on categorical classifications of flexibility, which may obscure functional relationships. This study is aimed at examining the associations between hamstring flexibility and multiple components of postural control in healthy young adults.

**Methods:**

This cross‐sectional observational study included 70 healthy young adults. Hamstring flexibility was assessed using the sit‐and‐reach test. Postural control was evaluated through dynamic balance (Y Balance Test), postural stability (Biodex Overall Stability Index), proprioception (joint position sense), and tactile acuity (two‐point discrimination). Between‐group comparisons and correlation analyses were performed. Multiple linear regression was conducted to identify independent predictors of dynamic balance performance.

**Results:**

No significant differences were observed between participants with and without hamstring shortness in dynamic balance, postural stability, proprioception, tactile acuity, or interlimb asymmetry (all *p* > 0.05). Hamstring flexibility was positively correlated with right‐limb YBT composite score (*r* = 0.298, *p* = 0.012), negatively correlated with Biodex Overall Stability Index (*r* = −0.241, *p* = 0.044), and weakly negatively correlated with shoulder joint position sense (*r* = −0.265, *p* = 0.027). In multiple linear regression analysis, hamstring flexibility remained an independent predictor of dynamic balance performance (*B* = 0.557, 95% CI 0.212–0.903, *p* = 0.002).

**Conclusions:**

Greater hamstring flexibility was associated with better dynamic balance performance and showed a weaker association with postural stability, whereas no robust associations were observed with sensory measures of postural control. These findings suggest that hamstring flexibility may be more closely related to motor than sensory aspects of postural control in healthy young adults. Furthermore, associations were identified when flexibility was analyzed as a continuous variable, highlighting the potential value of preserving continuous data in studies examining functional performance.

## 1. Introduction

Hamstring flexibility is an important component of musculoskeletal function and has been implicated in movement efficiency, lower extremity biomechanics, and postural control [1]. In particular, hamstring flexibility has been widely studied because of its role in lower extremity kinematics, pelvic alignment, and trunk stability during functional activities [2, 3]. Recent evidence suggests that hamstring tightness is associated with altered biomechanics, postural deviations, reduced range of motion, and functional limitations, highlighting the importance of maintaining adequate hamstring flexibility for optimal movement performance [4].

Sufficient hamstring flexibility has been associated with optimal lumbopelvic rhythm and efficient force transmission during dynamic tasks [5]. Conversely, reduced hamstring flexibility has been associated with altered movement patterns, restricted range of motion, and compensatory strategies that may affect dynamic balance performance [6]. Previous studies have suggested a potential relationship between lower extremity flexibility and dynamic balance performance; however, findings have been inconsistent across different populations and assessment methods [7, 8]. However, the extent to which hamstring flexibility contributes to different components of postural control remains incompletely understood [9].

Postural control is a multidimensional construct that involves integrating sensory and motor information for the purpose of maintaining stability [9]. Previous studies have suggested that muscle flexibility may influence proprioceptive acuity; however, findings have been inconsistent across studies [10, 11]. These inconsistencies may be attributable to differences in study populations, flexibility assessment methods, and the specific postural control outcomes evaluated. Consequently, it remains unclear whether hamstring flexibility primarily influences motor aspects of postural control, such as dynamic balance and global stability, or whether it also affects sensory domains, including joint position sense (JPS) and tactile acuity [12].

Another limitation of the present literature on flexibility is that many studies use categorical classifications (e.g., either having or not having hamstring shortness) to determine the effect of flexibility on function. Categorical approaches may not fully capture the complex and potentially graded relationship between flexibility and functional performance [13, 14]. Continuous analysis of flexibility may provide a more sensitive framework for detecting associations that are not evident in group‐based comparisons.

Therefore, this study is aimed at examining the associations between hamstring flexibility and both motor (dynamic balance and postural stability) and sensory (proprioception and tactile acuity) components of postural control in healthy young adults. We hypothesized that [1] hamstring flexibility would be associated with motor components of postural control, but not with sensory components, and [2] continuous measures of flexibility may reveal associations that are not apparent in categorical group comparisons.

## 2. Methods

### 2.1. Study Design and Participants

This cross‐sectional observational study was conducted in accordance with the STROBE guidelines at the Department of Physical Therapy and Rehabilitation, Bolu Abant İzzet Baysal University, Turkey. The study is aimed at examining the associations between hamstring flexibility and multiple components of postural control in healthy young adults. Data collection was performed between September 2025 and March 2026 in a controlled laboratory setting within the university.

The sample size was calculated based on both group comparisons and correlation analyses, considering the primary objectives of the study. For between‐group comparisons (participants with and without hamstring shortness), sample size estimation was performed assuming a large effect size (Cohen′s *d* = 0.80), in accordance with conventional effect size classifications used in G∗Power analyses [15]. Using a two‐tailed independent samples comparison with a significance level of *α* = 0.05 and statistical power of 80%, the minimum required sample size was estimated as 26 participants per group. Given that the primary analyses also included correlation‐based assessments, this sample size was considered sufficient to detect moderate correlations (*r* = 0.30), consistent with contemporary recommendations for correlation‐based study design and power estimation [16]. To account for potential dropouts, a 30% attrition rate was anticipated. Accordingly, the target sample size was increased to approximately 35 participants per group, resulting in a total sample size of 70 participants. All participants completed the study, and no data loss occurred.

**Figure 1:**
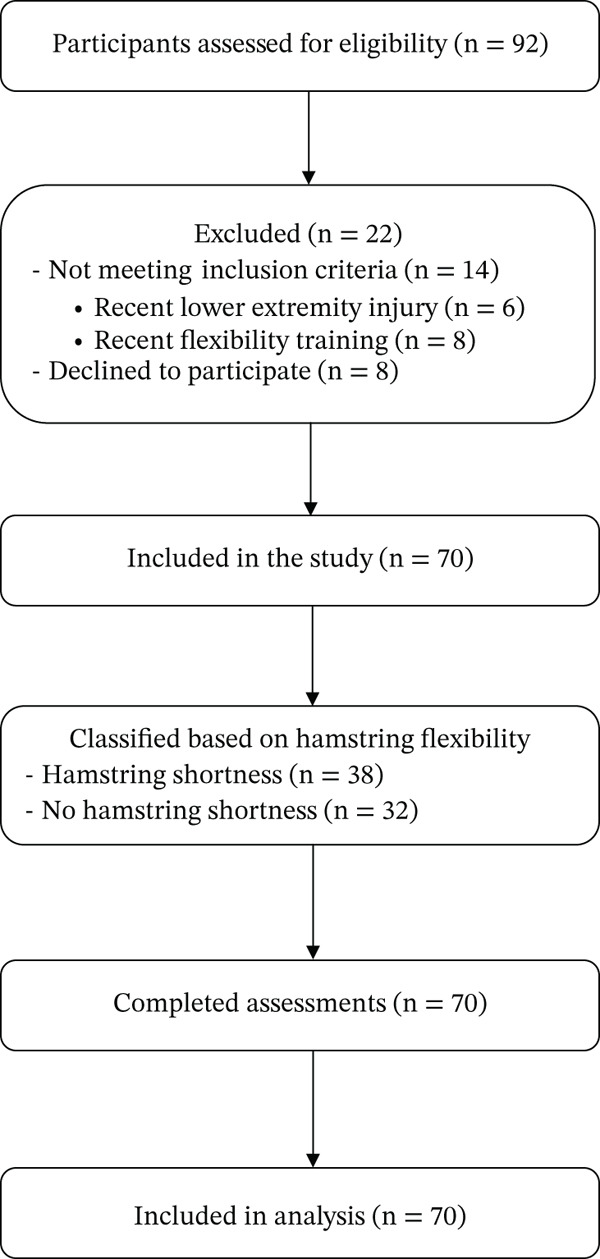
Flow diagram of participant recruitment, eligibility screening, and inclusion in the analysis.

The selection of participants for this study was completed via convenience sampling. The participants were recruited from student and staff populations at the university and had to be between 18 and 30 years old to qualify for the study. Individuals were excluded from participation in the study if they met any of the following criteria: (i) They had experienced an injury to their lower extremity or undergone lower extremity surgery within the previous 6 months; (ii) they had a neurological disorder, vestibular disorder, or musculoskeletal disorder, which would affect their balance; (iii) they had current pain at the time of the assessment; or (iv) they had engaged in a structured flexibility training program during the previous 3 months. To reduce selection bias, all eligible participants during the recruitment period were invited to participate, and standardized eligibility criteria were applied consistently. Informed consent was provided in writing by all participants prior to beginning the study. All aspects of this research complied with the Declaration of Helsinki concerning human subjects research. The participant recruitment process is illustrated in Figure 1.

### 2.2. Assessment Procedures

All assessments were performed by the same investigator. Participants were instructed to refrain from vigorous physical activity for at least 24 h prior to testing. Measurements were conducted in a single session, with adequate rest intervals provided between tests to minimize fatigue effects. All assessments were performed in the same predefined and standardized order for all participants. The testing sequence was not randomized.

#### 2.2.1. Hamstring Flexibility

**Figure 2:**
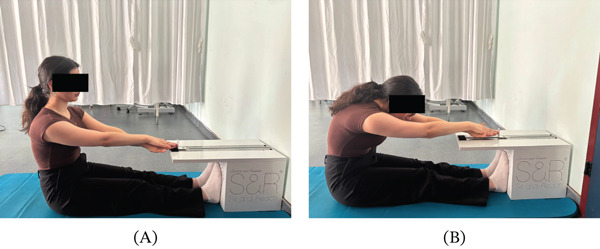
(A) Starting position. (B) Final reaching position. The participant was seated on the floor with the knees fully extended and the soles of the feet positioned against the sit‐and‐reach box. The participant then reached forward along the measuring scale, and the maximum distance achieved was recorded in centimeters.

Hamstring flexibility was assessed using the sit‐and‐reach test, a widely used method with established validity and excellent test–retest reliability (ICC = 0.98; 95% CI 0.97–0.99) for evaluating hamstring flexibility [17]. Assessment was performed using a standardized sit‐and‐reach box in accordance with the conventional sit‐and‐reach testing procedure. Participants were seated on the floor with their knees fully extended and the soles of their feet placed against the sit‐and‐reach box. They were instructed to reach forward with both hands as far as possible while maintaining full knee extension. The maximum distance achieved across three trials was recorded in centimeters. For exploratory between‐group comparisons, participants were categorized as having hamstring shortness or no hamstring shortness based on observational assessment during the sit‐and‐reach test. However, the primary analyses were conducted using continuous hamstring flexibility values. A representative photograph of the sit‐and‐reach testing setup is provided in Figure 2.

#### 2.2.2. Dynamic Balance

Dynamic balance performance was evaluated using the Y Balance Test (YBT), a measure of dynamic postural control with good to excellent reliability (ICC = 0.85–0.91; composite score ICC = 0.91) [18]. Prior to data collection, participants completed three familiarization trials in each direction to minimize potential learning effects. Subsequently, three recorded trials were performed. Participants performed reach tasks in the anterior, posteromedial, and posterolateral directions while maintaining single‐leg stance. Reach distances were normalized to leg length and expressed as percentages [19]. Reach distances were recorded in inches and converted to centimeters prior to normalization. The composite score was calculated by averaging the three planes of reach. To evaluate interlimb symmetry, asymmetry values were calculated by determining the absolute difference between limbs [20].

#### 2.2.3. Global Postural Stability

Global postural stability was assessed using the Biodex Balance System (Biodex Medical Systems, Shirley, New York, United States) [21]. All assessments were performed with the platform set at stability Level 8. The Biodex Balance System has demonstrated excellent reliability for the Overall Stability Index (OSI) (ICC = 0.93; 95% CI 0.83–0.97) [22]. Participants performed a static balance test under standardized conditions. The OSI was recorded as the primary outcome, with higher values indicating poorer postural stability. In addition, sway‐related measures were recorded as indicators of postural control performance [23].

#### 2.2.4. Proprioception

Proprioceptive accuracy was assessed using JPS, a widely accepted measure of proprioceptive function, in both the knee and shoulder joints [9, 24]. A previous study demonstrated good test–retest reliability for shoulder JPS assessment (ICC = 0.78; 95% CI 0.57–0.89) [25]. Shoulder JPS was included to evaluate proprioceptive function at a body region anatomically distant from the hamstrings. This approach allowed exploration of whether hamstring flexibility was associated with generalized proprioceptive accuracy rather than solely local lower extremity function. For the knee JPS assessment, participants were positioned prone on an examination table with the knee initially in full extension (0°). The lower leg was then passively moved to 45° of knee flexion, which served as the target position. For the shoulder JPS assessment, participants were seated with the arm alongside the body (0° abduction), and the arm was passively moved to 90° of shoulder abduction, which served as the target position. Participants were subsequently asked to actively reproduce the target positions. The absolute angular distance in degrees between the target and reproduced positions was recorded, with lower values reflecting greater proprioceptive accuracy [9].

#### 2.2.5. Tactile Acuity

Tactile acuity was evaluated using the two‐point discrimination (2PD) test, a widely used method for assessing cutaneous sensory discrimination [26]. The static 2PD test has demonstrated excellent interrater reliability (ICC = 0.99; lower 95% CI = 0.98) [27]. The 2PD test was assessed at the fingertip because the fingertip is the most commonly used and most sensitive site for assessing tactile spatial discrimination. Measurements were performed using a discriminator wheel (Baseline, Fabrication Enterprises Inc., White Plains, New York, United States). The smallest distance at which two points were perceived as distinct was recorded in millimeters, with lower values indicating better tactile discrimination.

### 2.3. Statistical Analysis

All statistical analyses were performed using R software (R Foundation for Statistical Computing, Vienna, Austria). The significance level was set at *p* < 0.05. Normality of the data was assessed using the Shapiro–Wilk test. Continuous variables with normal distribution were expressed as mean ± standard deviation, whereas nonnormally distributed variables were presented as median and interquartile range. Between‐group comparisons were performed using independent samples *t*‐tests for normally distributed variables and Mann–Whitney *U* tests for nonnormally distributed variables. Categorical variables were analyzed using the chi‐square test or Fisher′s exact test when appropriate. Spearman correlation analysis was used to examine associations between hamstring flexibility and outcome variables. Multiple linear regression analysis was performed for the primary outcome (YBT composite score of the right limb), including hamstring flexibility, BMI, and sex as predictors. Exploratory analyses were conducted for the composite sway index; however, this variable was not included in the primary analysis because of lack of statistical significance. Prior to regression analyses, model assumptions were assessed by examining residual distributions, homogeneity of variance, and influential observations. No major violations of regression assumptions were identified. Only right‐limb measurements were included in regression analyses to avoid statistical dependence between limbs and to ensure a standardized approach across participants.

## 3. Results

### 3.1. Participant Characteristics

A total of 92 individuals were assessed for eligibility. Of these, 22 were excluded because of not meeting the inclusion criteria or declining participation. Finally, 70 participants were included in the study and completed all assessments. Baseline demographic and anthropometric characteristics are presented in Table 1. There were no statistical differences of age, height, weight, body mass index, gender, dominance, or sport experience between groups (all *p* > 0.05). As expected, hamstring flexibility was significantly lower in the hamstring shortness group compared with the nonshortness group (21.4 ± 6.43 cm vs. 30.2 ± 7.49 cm, *p* < 0.001).

**Table 1:** Baseline and outcome characteristics of participants according to hamstring shortness.

Variable	No hamstring shortness (*n* = 32)	Hamstring shortness (*n* = 38)	*p*
Age (years)	21.6 ± 1.87	22.0 ± 2.03	0.381
Height (cm)	168.9 ± 10.2	167.6 ± 8.88	0.575
Weight (kg)	66.3 ± 12.8	63.0 ± 11.1	0.258
BMI (kg/m^2^)	23.1 ± 3.50	22.3 ± 2.75	0.290
Sex (female/male)	20/12	28/10	0.456
Dominant hand (right/left)	29/3	36/2	0.654^a^
Sport experience (yes/no)	4/28	8/30	0.526^a^
Hamstring flexibility (cm)	30.2 ± 7.49	21.4 ± 6.43	< 0.001
JPS shoulder (right, °)	2.30 (0.93–4.00)	3.50 (1.30–5.30)	0.261
YBT composite score (right, %)	61.0 ± 13.5	55.4 ± 12.9	0.082
YBT composite score (left, %)	60.6 ± 13.0	55.1 ± 13.1	0.082
YBT composite asymmetry (%)	3.97 ± 2.87	4.97 ± 4.55	0.465
Biodex OSI	0.80 (0.60–1.10)	0.90 (0.63–1.28)	0.225
JPS knee (right, °)	5.00 (3.22–7.85)	5.85 (3.40–8.82)	0.363
2PD finger (mm)	3.59 ± 0.77	3.81 ± 0.82	0.291

*Note:* Data are presented as mean ± standard deviation, median (interquartile range), or number (*n*). Independent samples *t*‐test was used for normally distributed variables, and Mann–Whitney *U* test was used for nonnormally distributed variables.

^a^Fisher′s exact test.

### 3.2. Between‐Group Comparisons

Between‐group comparisons of all outcome measures are presented in Table 1. No statistically significant differences were observed in dynamic balance performance between participants with and without hamstring shortness. The right‐limb YBT composite score was 61.0 ± 13.5 in participants without hamstring shortness and 55.4 ± 12.9 in those with hamstring shortness (*p* = 0.082). Similarly, the left‐limb YBT composite score was 60.6 ± 13.0 and 55.1 ± 13.1, respectively (*p* = 0.082). YBT composite asymmetry was also comparable between groups (3.97 ± 2.87 vs. 4.97 ± 4.55, *p* = 0.465).

Global postural stability, assessed using the OSI, was also comparable between groups (*p* = 0.225). No significant between‐group differences were observed for proprioceptive accuracy (knee JPS error, *p* = 0.363) or tactile acuity (2PD, *p* = 0.291).

### 3.3. Correlation Analyses

Results from the unadjusted correlation analyses indicated significant relationships between hamstring flexibility and selected motor components of postural control (Table 2). Specifically, a positive correlation was observed between hamstring flexibility and the right‐limb YBT composite score (*r* = 0.298, *p* = 0.012), whereas a negative correlation was found between hamstring flexibility and the OSI (*r* = −0.241, *p* = 0.044). A weak negative correlation was also observed between hamstring flexibility and shoulder JPS (*r* = −0.265, *p* = 0.027). These findings suggest that greater hamstring flexibility is associated with better dynamic balance performance and lower postural instability, with a weak association also observed for shoulder JPS.

**Table 2:** Correlations between hamstring flexibility and outcome measures.

Variable	*r*	*p*
YBT composite score (right)	0.298	0.012
YBT composite asymmetry	−0.144	0.236
Biodex OSI	−0.241	0.044
Knee joint position sense error (right)	−0.138	0.255
Shoulder joint position sense error (right)	−0.265	0.027
Two‐point discrimination, mean finger score	−0.036	0.765

*Note:* Spearman correlation analysis was used to examine the association between hamstring flexibility and outcome variables.

**Figure 3:**
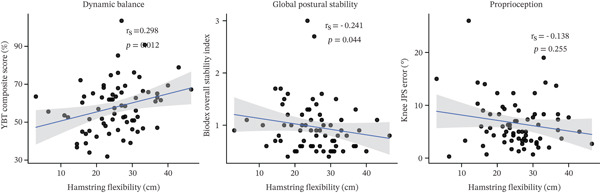
Relationships between hamstring flexibility and postural control outcomes. (A) Hamstring flexibility was positively associated with Y Balance Test (YBT) composite score. (B) A weak inverse association was observed between hamstring flexibility and Biodex Overall Stability Index. (C) No significant association was observed between hamstring flexibility and knee joint position sense (JPS) error. Solid lines represent fitted regression lines, and shaded areas represent 95% confidence intervals. The fitted regression lines are shown for visual guidance only and do not represent the Spearman rank correlation analysis.

These relationships are illustrated in Figure 3. As shown in Figure 3A, greater hamstring flexibility was associated with higher YBT composite scores (*r* = 0.298, *p* = 0.012). Multiple linear regression analysis further demonstrated that hamstring flexibility was a significant positive predictor of YBT composite performance (*B* = 0.557, 95% CI 0.212–0.903, *p* = 0.002). Similarly, Figure 3B demonstrates a weak inverse association between hamstring flexibility and OSI values (*r* = −0.241, *p* = 0.044). However, this association was not confirmed in the corresponding linear regression analysis (*B* = −0.010, 95% CI −0.024 to 0.003, *p* = 0.134). In contrast, hamstring flexibility was not significantly associated with knee JPS error (*r* = −0.138, *p* = 0.255), as illustrated in Figure 3C. No significant associations were observed between hamstring flexibility and YBT composite asymmetry (*r* = −0.144, *p* = 0.236) or 2PD (*r* = −0.036, *p* = 0.765).

### 3.4. Regression Analyses

In adjusted analyses, multiple linear regression analysis was performed to determine whether hamstring flexibility independently predicted dynamic balance performance (Table 3). In the adjusted model, hamstring flexibility was a significant independent predictor of right‐limb YBT composite score (*B* = 0.557, 95% CI 0.212–0.903, *p* = 0.002). Male sex was also identified as a significant predictor (*B* = 12.358, 95% CI 6.279–18.436, *p* < 0.001), whereas body mass index was not significantly associated with dynamic balance performance (*B* = 0.229, 95% CI −0.677 to 1.135, *p* = 0.615). The overall model was statistically significant (*F* = 8.434, *p* < 0.001) and explained 27.7% of the variance in dynamic balance performance (*R* ^2^ = 0.277; adjusted *R* ^2^ = 0.244). In practical terms, each 1‐cm increase in hamstring flexibility was associated with an approximately 0.56‐point increase in YBT composite score.

**Table 3:** Multiple linear regression analysis for YBT composite score (right).

Predictor	Regression coefficient (*B*)	95% CI	*p*
Intercept	34.731	12.348–57.113	0.003
Hamstring flexibility (cm)	0.557	0.212–0.903	0.002
BMI (kg/m^2^)	0.229	−0.677 to 1.135	0.615
Male sex	12.358	6.279–18.436	< 0.001

*Note:* Model summary: *R* ^2^ = 0.277, adjusted *R* ^2^ = 0.244, *F*(3, 66) = 8.434, *p* < 0.001.

### 3.5. Exploratory Analyses

Table S1 presents the exploratory analyses of the sway composite index, including its correlations with hamstring flexibility and the OSI. A moderate positive correlation was observed between the OSI and sway composite score (*r* = 0.383, *p* = 0.001), indicating consistency between these two measures of postural stability. No significant correlation was identified between hamstring flexibility and sway composite score (*r* = −0.206, *p* = 0.087).

Categorical comparison of hamstring shortness did not yield significant differences in postural control measures. However, analyses based on continuous data demonstrated that greater hamstring flexibility was associated with better dynamic balance performance and showed a weak association with overall postural stability. These relationships were limited to motor components of postural control and were not observed in sensory domains.

## 4. Discussion

This study investigated the association of hamstring flexibility with multiple components of postural control, including dynamic balance, global postural stability, proprioceptive accuracy, tactile discrimination, and interlimb asymmetry. Recent findings have highlighted that reduced hamstring flexibility is associated with altered biomechanics, postural deviations, reduced range of motion, and functional limitations, further supporting the functional importance of maintaining adequate hamstring flexibility [4]. Our findings indicate that greater hamstring flexibility is significantly associated with better dynamic balance performance and shows a weak association with global postural stability, whereas no significant associations were observed with proprioceptive accuracy, tactile discrimination, or asymmetry measures. These findings are consistent with previous studies demonstrating associations between hamstring flexibility, lower extremity kinematics, and lumbopelvic rhythm [2, 28]. However, in contrast to studies suggesting that dynamic balance performance is more strongly related to muscle strength, our findings indicate that flexibility may independently contribute to motor performance [29]. Furthermore, the discrepancy between categorical and continuous analyses suggests that flexibility may be better interpreted along a continuum rather than as a binary characteristic. This interpretation is consistent with current concepts in biomechanics and exercise physiology, which emphasize dose–response relationships between physical attributes and functional performance [13].

The significant positive association between hamstring flexibility and dynamic balance performance is consistent with the biomechanical role of the hamstrings in regulating hip flexion, pelvic positioning, and trunk alignment. The current findings are consistent with previous research suggesting that adequate hamstring flexibility is associated with optimal lumbopelvic rhythm, which may contribute to better functional stability and dynamic balance performance [3]. Dynamic balance appears to relate more closely to hip muscle strength than to hip muscle flexibility based on some studies [29]. This discrepancy may be explained by methodological differences, as prior research often relied on categorical approaches or injury‐based populations, whereas the present study used continuous analyses. The YBT is sensitive to multiple factors, including strength, range of motion, and lumbopelvic coordination, which may explain the observed association with hamstring flexibility. From a biomechanical perspective, increased hamstring flexibility may reduce passive stiffness in the posterior chain, allowing greater hip flexion and lumbopelvic motion during reach tasks. This may facilitate longer reach distances and contribute to improved YBT performance. Although hamstring flexibility remained a significant predictor after adjustment for sex and BMI, sex was the strongest predictor in the model and the overall explained variance was modest. Therefore, the observed association should be interpreted cautiously, as other unmeasured factors, particularly lower extremity muscle strength, may also contribute to YBT performance. Although the observed correlation was modest in magnitude (*r* = 0.298), the regression analysis suggests a potentially meaningful functional relationship.

A negative association was observed between OSI and hamstring flexibility, indicating that individuals with greater flexibility tended to exhibit lower OSI values and, therefore, better overall postural stability. However, this relationship was modest in magnitude and was not retained in the adjusted regression analysis. Therefore, the clinical relevance of the association between hamstring flexibility and global postural stability appears more limited than that observed for dynamic balance performance. One possible explanation is that greater hamstring flexibility may reduce passive resistance within the posterior kinetic chain, facilitating more efficient load distribution and allowing finer corrective adjustments during balance control. Previous research has similarly suggested that adequate muscle–tendon extensibility may contribute to more effective regulation of the center of mass during postural tasks [30]. Taken together with the YBT findings, these results suggest that hamstring flexibility may be more strongly related to motor aspects of postural control than to sensory components. Overall, the present findings support a potential role of hamstring flexibility in dynamic balance performance, whereas its contribution to global postural stability appears weaker and should be interpreted with caution.

Unlike the dynamic balance performance and stability results, no significant association was observed between hamstring flexibility and knee JPS. Interestingly, a weak negative correlation was identified between hamstring flexibility and shoulder JPS error. However, this finding should be interpreted cautiously given the modest effect size, multiple correlation testing, and the exploratory nature of this analysis. Overall, these findings do not provide strong evidence that hamstring flexibility substantially influences proprioceptive accuracy. JPS primarily reflects the ability to perceive and reproduce joint angles and is largely dependent on mechanoreceptor function and the quality of afferent neural input rather than muscle extensibility itself. Previous literature also emphasizes the sensory‐driven nature of proprioception [9, 24]. Accordingly, the lack of association observed in this study indicates that improvements in flexibility alone may not directly enhance proprioceptive function. From a clinical perspective, these findings indicate that flexibility‐focused interventions may not be sufficient to improve sensorimotor performance and may need to be combined with targeted proprioceptive and neuromuscular training strategies.

Similarly, no significant association was found between hamstring flexibility and tactile acuity assessed by the 2PD test. In terms of postural regulation, tactile feedback is known to assist with postural control, particularly when visual input is reduced. However, the absence of a significant association between hamstring flexibility and tactile acuity in the present study suggests that tactile acuity may be less relevant to dynamic balance performance tasks. The absence of associations in both proprioceptive and tactile domains suggests that hamstring flexibility primarily influences motor control mechanisms rather than sensory processing pathways. This view supports theories that state that sensory input and motor output elements of postural control are regulated by separate physiologic processes [31]. Accordingly, the observed association between greater hamstring flexibility and dynamic balance performance may be more closely related to mechanical and biomechanical factors than to peripheral sensory sensitivity.

Analysis of interlimb asymmetry revealed that there is no relationship between hamstring flexibility and any measure of asymmetry. Previous evidence suggests that asymmetry metrics may be more strongly influenced by factors such as injury history, limb dominance, and specific neuromuscular deficits [20]. In contrast, hamstring flexibility may be less relevant to side‐to‐side performance differences than to overall movement capacity. Consequently, hamstring flexibility appears to improve functional capacity rather than remediate side‐to‐side asymmetries. This finding is consistent with the notion that flexibility is a relatively global physical characteristic and may not be a primary determinant of interlimb asymmetry. From a clinical perspective, these results suggest that flexibility‐focused interventions alone may be insufficient to address substantial asymmetries and may need to be complemented by targeted strength, coordination, and balance training approaches aimed at restoring limb symmetry [32].

One methodological observation from the present study was the difference between the results obtained from categorical and continuous analyses. Although no significant differences were observed when participants were classified according to the presence or absence of hamstring shortness, significant associations emerged when hamstring flexibility was analyzed as a continuous variable. However, comparison of effect size estimates demonstrated generally similar magnitudes across analytical approaches (Table S2). Therefore, the present findings should not be interpreted as evidence that one analytical approach is inherently superior to the other. Rather, the results suggest that continuous flexibility measures may provide additional information regarding functional performance and may detect associations that are not apparent in categorical group comparisons. In addition, the nonsignificant categorical findings may partly reflect reduced statistical power associated with dichotomization in a relatively modest sample [13, 14].

The exploratory analysis did not demonstrate a significant association between hamstring flexibility and the sway composite index. In contrast, a significant correlation was observed between hamstring flexibility and the OSI, suggesting that different balance measures may capture distinct aspects of postural control. Previous literature has similarly highlighted that instrumental balance measures do not necessarily reflect the same components of postural stability because of differences in measurement protocols and underlying constructs [33]. Accordingly, the present findings suggest that OSI may be more responsive to flexibility‐related characteristics than composite sway indices, although this interpretation should be considered exploratory. Further research is needed to clarify the relative sensitivity of different balance parameters to flexibility‐related adaptations.

From a clinical perspective, the present findings suggest that hamstring flexibility may be relevant to dynamic balance performance and, to a lesser extent, global postural stability in healthy young adults. Given the importance of balance for functional performance and injury prevention, flexibility training may represent a useful component of rehabilitation and fitness programs aimed at optimizing movement efficiency and postural control. However, the absence of significant associations with proprioception, tactile acuity, and interlimb asymmetry suggests that the relationship between hamstring flexibility and postural control may vary across different motor and sensory domains. Therefore, flexibility‐focused interventions should be complemented by targeted balance, proprioceptive, and neuromuscular training strategies to optimize sensorimotor function and overall movement performance [34].

This study has several limitations. First, the cross‐sectional design precludes causal inference, and it cannot be determined whether increased flexibility directly leads to improved dynamic balance performance. Second, study participants were healthy young adults, and therefore, results may not be representative of clinical or older patient populations. Participants were recruited using convenience sampling from a single university population, which may have introduced selection bias and further limits the external validity of the findings. Third, although multiple domains of postural control were assessed, other relevant factors such as muscle strength, coordination, and central nervous system integration were not directly measured. In particular, lower extremity muscle strength is a well‐established determinant of YBT performance and may have confounded the observed association between hamstring flexibility and dynamic balance performance. Furthermore, habitual physical activity was not quantified using a validated physical activity measure. Although sport experience was recorded, this binary variable may not adequately reflect overall physical activity levels. Therefore, residual confounding related to physical activity cannot be excluded. In addition, postural control is a multidimensional construct, and no single assessment tool can fully capture all of its components. Therefore, measurement error and construct validity limitations should also be considered when interpreting the findings. In addition, the categorical classification of hamstring shortness was based on observational assessment rather than predefined normative thresholds, and therefore, the results of between‐group comparisons should be interpreted with caution. In addition, multiple correlation analyses were performed, and no formal adjustment for multiple comparisons was applied. Therefore, residual confounding from unmeasured factors cannot be excluded, and the reported associations should be interpreted with appropriate caution. Finally, the use of field‐based flexibility assessment methods, although practical, may not fully capture the complexity of muscle–tendon properties.

## 5. Conclusıon

In conclusion, greater hamstring flexibility was associated with better dynamic balance performance and showed a weaker association with postural stability, but not with proprioception, tactile discrimination, or interlimb asymmetry. These findings suggest that the relationship between hamstring flexibility and postural control may be more closely related to biomechanical factors than to sensory factors. Although categorical group comparisons were not statistically significant, continuous analyses identified associations between hamstring flexibility and selected postural control outcomes. These findings suggest that continuous flexibility measures may provide additional information regarding functional performance; however, they do not establish the superiority of one analytical approach over another.

**Nomenclature**
2PD: two‐point discrimination
BMI: body mass index
CI: confidence interval
JPS: joint position sense
OSI: Overall Stability Index
*R* ^2^
: coefficient of determination
SD: standard deviation
YBT: Y Balance Test

## Author Contributions

A.Ö., Ş.Ş., and S.A. contributed to the conceptualization and methodology of the study. A.Ö. performed the formal analysis. A.Ö., Ş.Ş., and S.A. drafted the manuscript and contributed to its critical revision.

## Funding

This study was supported by the Bolu Abant İzzet Baysal University Scientific Research Projects Coordination Unit (2025‐ÖĞP‐006).

## Disclosure

All authors read and approved the final manuscript.

## Ethics Statement

The study was conducted in accordance with the principles of the Declaration of Helsinki. Ethical approval was obtained from the Non‐Interventional Clinical Research Ethics Committee of Bolu Abant İzzet Baysal University (Approval No: 2025/233, Date: June 3, 2025). All participants provided written informed consent prior to participation, and all data were anonymized before analysis.

## Consent

The authors have nothing to report.

## Conflicts of Interest

The authors declare no conflicts of interest.

## Supporting information


**Supporting Information** Additional supporting information can be found online in the Supporting Information section. Table S1: Exploratory correlations involving sway composite index, hamstring flexibility, and OSI. Table S2: Comparison of effect size estimates obtained from categorical and continuous analyses of hamstring flexibility.

## Data Availability

The datasets generated and/or analyzed during the current study are available in the Zenodo repository, 10.5281/zenodo.19334044.

## References

[1] Behm D. G. , Blazevich A. J. , Kay A. D. , and McHugh M. , Acute Effects of Muscle Stretching on Physical Performance, Range of Motion, and Injury Incidence in Healthy Active Individuals: A Systematic Review, Applied Physiology, Nutrition, and Metabolism. (2016) 41, no. 1, 1–11, 10.1139/apnm-2015-0235, 26642915. 26642915

[2] Freitas S. R. , Mendes B. , Le Sant G. , Andrade R. J. , Nordez A. , and Milanovic Z. , Can Chronic Stretching Change the Muscle-Tendon Mechanical Properties? A Review, Scandinavian Journal of Medicine & Science in Sports. (2018) 28, no. 3, 794–806, 10.1111/sms.12957, 28801950. 28801950

[3] Moreside J. M. and McGill S. M. , Improvements in Hip Flexibility Do Not Transfer to Mobility in Functional Movement Patterns, Journal of Strength and Conditioning Research. (2013) 27, no. 10, 2635–2643, 10.1519/JSC.0b013e318295d521, 23591949. 23591949

[4] Miçooğulları M. , Özgökalp İ. , and Angın S. , Effects of Instrument Assisted and Functional Soft Tissue Mobilization on Hamstring Flexibility and Skinfold Thickness in Sedentary Adults, Scientific Reports. (2026) 16, no. 1, 10.1038/s41598-026-36856-w, 41571821. PMC12902047 41571821

[5] Chumanov E. S. , Heiderscheit B. C. , and Thelen D. G. , Hamstring Musculotendon Dynamics During Stance and Swing Phases of High-Speed Running, Medicine and Science in Sports and Exercise. (2011) 43, no. 3, 525–532, 10.1249/MSS.0b013e3181f23fe8, 20689454. 20689454 PMC3057086

[6] Kay A. D. and Blazevich A. J. , Effect of Acute Static Stretch on Maximal Muscle Performance, Medicine and Science in Sports and Exercise. (2012) 44, no. 1, 154–164, 10.1249/MSS.0b013e318225cb27. 21659901

[7] Encarnación-Martínez A. , García-Gallart A. , Pérez-Soriano P. , Catalá-Vilaplana I. , Rizo-Albero J. , and Sanchis-Sanchis R. , Effect of Hamstring Tightness and Fatigue on Dynamic Stability and Agility in Physically Active Young Men, Sensors. (2023) 23, no. 3, 10.3390/s23031633, 36772673. PMC9921967 36772673

[8] Neji Z. , Attia A. , Negra Y. , Sammoud S. , Bouguezzi R. , and Hachana Y. , Evaluation of Reliability and Sensitivity of the Y-Balance Test for Quantifying Lower Limb Asymmetry in Prepubescent Soccer and Basketball Players, Journal of Physical Education and Sport. (2020) 20, no. 4, 1823–1831.

[9] Proske U. and Gandevia S. C. , The Proprioceptive Senses: Their Roles in Signaling Body Shape, Body Position and Movement, and Muscle Force, Physiological Reviews. (2012) 92, no. 4, 1651–1697, 10.1152/physrev.00048.2011, 23073629. 23073629

[10] Konrad A. and Tilp M. , Increased Range of Motion After Static Stretching Is Not due to Changes in Muscle and Tendon Structures, Clinical Biomechanics. (2014) 29, no. 6, 636–642, 10.1016/j.clinbiomech.2014.04.013. 24856792

[11] Simic L. , Sarabon N. , and Markovic G. , Does Pre-Exercise Static Stretching Inhibit Maximal Muscular Performance? A Meta-Analytical Review, Scandinavian Journal of Medicine & Science in Sports. (2013) 23, no. 2, 131–148, 10.1111/j.1600-0838.2012.01444.x, 22316148. 22316148

[12] Horak F. B. , Postural Orientation and Equilibrium: What Do We Need to Know About Neural Control of Balance to Prevent Falls?, Age and Ageing. (2006) 35, no. supplement_2, ii7–ii11, 10.1093/ageing/afl077, 16926210. 16926210

[13] Altman D. G. and Royston P. , The Cost of Dichotomising Continuous Variables, British Medical Journal. (2006) 332, no. 7549, 16675816. 10.1136/bmj.332.7549.1080 PMC1458573 16675816

[14] Royston P. , Altman D. G. , and Sauerbrei W. , Dichotomizing Continuous Predictors in Multiple Regression: A Bad Idea, Statistics in Medicine. (2006) 25, no. 1, 127–141, 10.1002/sim.2331, 16217841. 16217841

[15] Negida A. , Sample Size Calculation Guide - Part 7: How to Calculate the Sample Size Based on a Correlation, Advanced Journal of Emergency Medicine. (2020) 4, no. 2, 10.22114/ajem.v0i0.344, 32322802. PMC7163254 32322802

[16] Kang H. , Sample Size Determination and Power Analysis Using the G∗Power Software, Journal of Educational Evaluation for Health Professions. (2021) 18, 10.3352/jeehp.2021.18.17, 34325496. PMC8441096 34325496

[17] Annino G. , Alashram A. , Romagnoli C. , Balducci E. , De Paolis M. , Manzi V. , and Padua E. , Acute Effects of Kinesio Taping on Functional Performance in Healthy Soccer Players: A Randomized, Controlled Crossover Trial, Journal of Functional Morphology and Kinesiology. (2023) 8, no. 1, 10.3390/jfmk8010002. PMC9844374 36648894

[18] Aktar H. , Muskan P. , Amjad R. , Satti A. K. , Javed M. , and Shahid M. , Correlation of Hamstring Tightness With Balance and Mobility in Young Adults, Pakistan Journal of Medical and Health Sciences. (2023) 17, no. 3, 295–297, 10.53350/pjmhs2023173295.

[19] Shaffer S. W. , Teyhen D. S. , Lorenson C. L. , Warren R. L. , Koreerat C. M. , Straseske C. A. , and Childs J. D. , Y-Balance Test: A Reliability Study Involving Multiple Raters, Military Medicine. (2013) 178, no. 11, 1264–1270, 10.7205/MILMED-D-13-00222, 24183777. 24183777

[20] Bishop C. , Turner A. , and Read P. , Effects of Inter-Limb Asymmetries on Physical and Sports Performance: A Systematic Review, Journal of Sports Sciences. (2018) 36, no. 10, 1135–1144, 10.1080/02640414.2017.1361894, 28767317. 28767317

[21] Cachupe W. J. C. , Shifflett B. , Kahanov L. , and Wughalter E. H. , Reliability of Biodex Balance System Measures, Measurement in Physical Education and Exercise Science. (2001) 5, no. 2, 97–108, 10.1207/S15327841MPEE0502_3.

[22] Pereira H. M. , Campos T. F. , Santos M. B. , Cardoso J. R. , Garcia M. C. , and Cohen M. , Influence of Knee Position on the Postural Stability Index Registered by the Biodex Stability System, Gait & Posture. (2008) 28, no. 4, 668–672, 10.1016/j.gaitpost.2008.05.003, 18573663. 18573663

[23] Quijoux F. , Nicolaï A. , Chairi I. , Bargiotas I. , Ricard D. , Yelnik A. , Oudre L. , Bertin-Hugault F. , Vidal P. P. , Vayatis N. , Buffat S. , and Audiffren J. , A Review of Center of Pressure (COP) Variables to Quantify Standing Balance in Elderly People: Algorithms and Open-Access Code, Physiological Reports. (2021) 9, no. 22, e15067, 10.14814/phy2.15067, 34826208. 34826208 PMC8623280

[24] Han J. , Waddington G. , Adams R. , Anson J. , and Liu Y. , Assessing Proprioception: A Critical Review of Methods, Journal of Sport and Health Science. (2016) 5, no. 1, 80–90, 10.1016/j.jshs.2014.10.004, 30356896. 30356896 PMC6191985

[25] Sutton P. , Lund Ohlsson M. , and Röijezon U. , Reduced Shoulder Proprioception due to Fatigue After Repeated Handball Throws and Evaluation of Test-Retest Reliability of a Clinical Shoulder Joint Position Test, Shoulder & Elbow. (2024) 16, no. 1_supplement, 100–109, 10.1177/17585732221139795, 38425739. 38425739 PMC10901175

[26] Tong J. , Mao O. , and Goldreich D. , Two-Point Orientation Discrimination Versus the Traditional Two-Point Test for Tactile Spatial Acuity Assessment, Frontiers in Human Neuroscience. (2013) 7, 10.3389/fnhum.2013.00579, 24062677. PMC3772339 24062677

[27] Novak C. B. , Mackinnon S. E. , Williams J. I. , and Kelly L. , Establishment of Reliability in the Evaluation of Hand Sensibility, Plastic and Reconstructive Surgery. (1993) 92, no. 2, 311–322, 10.1097/00006534-199308000-00017, 8337282. 8337282

[28] Fukaya T. , Sato S. , Yahata K. , Yoshida R. , Takeuchi K. , and Nakamura M. , Effects of Stretching Intensity on Range of Motion and Muscle Stiffness: A Narrative Review, Journal of Bodywork and Movement Therapies. (2022) 32, 68–76, 10.1016/j.jbmt.2022.04.011, 36180161. 36180161

[29] Gribble P. A. , Hertel J. , and Plisky P. , Using the Star Excursion Balance Test to Assess Dynamic Postural-Control Deficits and Outcomes in Lower Extremity Injury: A Literature and Systematic Review, Journal of Athletic Training. (2012) 47, no. 3, 339–357, 10.4085/1062-6050-47.3.08, 22892416. 22892416 PMC3392165

[30] Granacher U. , Gollhofer A. , Hortobágyi T. , Kressig R. W. , and Muehlbauer T. , The Importance of Trunk Muscle Strength for Balance, Functional Performance, and Fall Prevention in Seniors: A Systematic Review, Sports Medicine. (2013) 43, no. 7, 627–641, 10.1007/s40279-013-0041-1, 23568373. 23568373

[31] Viseux F. J. F. , The Sensory Role of the Sole of the Foot: Review and Update on Clinical perspectives, Clinical Neurophysiology. (2020) 50, no. 1, 55–68, 10.1016/j.neucli.2019.12.003, 32007381. 32007381

[32] Hewett T. E. , Myer G. D. , Ford K. R. , Heidt R. S. , Colosimo A. J. , McLean S. G. , van den Bogert A. J. , Paterno M. V. , and Succop P. , Biomechanical Measures of Neuromuscular Control and Valgus Loading of the Knee Predict Anterior Cruciate Ligament Injury Risk in Female Athletes: A Prospective Study, American Journal of Sports Medicine. (2005) 33, no. 4, 492–501, 10.1177/0363546504269591, 15722287. 15722287

[33] Pau M. , Leban B. , Collu G. , and Migliaccio G. M. , Effect of Light and Vigorous Physical Activity on Balance and Gait of Older Adults, Archives of Gerontology and Geriatrics. (2014) 59, no. 3, 568–573, 10.1016/j.archger.2014.07.008, 25127848. 25127848

[34] Zemková E. , Sport-Specific Balance, Sports Medicine. (2014) 44, no. 5, 579–590, 10.1007/s40279-013-0130-1. 24293269

