# Supplementary material for: Beyond Hamstring Shortness: Continuous Flexibility Is Associated With Balance and Stability but Not Sensory Measures—A Cross‐Sectional Study
Source: ScientificWorldJournal. 2026 Aug 13;2026:5729491. doi: 10.1155/tswj/5729491 (PMC13473833; doi:10.1155/tswj/5729491)
Supplement: Supplementary file 1 [file TSWJ-2026-5729491-s001.docx]

**Supplementary Table S1.** Exploratory correlations involving sway composite index, hamstring flexibility, and OSI.

| **Variable** | **r** | **p** |
| --- | --- | --- |
| Hamstring flexibility vs sway composite | -0.206 | 0.087 |
| OSI vs sway composite | 0.383 | 0.001 |

r, Spearman’s rank correlation coefficient; OSI, Overall Stability Index.

**Supplementary Table S2.** Comparison of effect size estimates obtained from categorical and continuous analyses of hamstring flexibility.

| **Outcome** | **d** | **r** |
| --- | --- | --- |
| **YBT** | 0.35 | 0.298 |
| **OSI** | -0.27 | -0.241 |
| **Knee JPS** | -0.31 | -0.138 |
| **Shoulder JPS** | -0.27 | -0.265 |
| **2PD** | -0.27 | -0.036 |

Cohen's d represents effect sizes for categorical group comparisons, whereas Spearman's rank correlation coefficient (r) represents the strength and direction of associations in continuous analyses. Effect size estimates are provided to aid interpretation of findings across analytical approaches.
